# Phase II study of S-1, a novel oral fluorouracil, in advanced non-small-cell lung cancer

**DOI:** 10.1054/bjoc.2001.2031

**Published:** 2001-09

**Authors:** M Kawahara, K Furuse, Y Segawa, K Yoshimori, K Matsui, S Kudoh, K Hasegawa, H Niitani, S-1 Cooperative Study Group (Lung Cancer Working Group)

**Affiliations:** Department of Internal Medicine, National Kinki Central Hospital for Chest Diseases, Osaka; Department of Internal Medicine, National Shikoku Cancer Center Hospital, Matuyama; Department of Respiratory Organs, Japan Anti-Tuberculosis Association Fukujuji Hospital, Tokyo; Second Department of Internal Medicine, Osaka Prefectural Habikino Hospital, Osaka; Fourth Department of Internal Medicine, Nippon Medical School, Tokyo; Department of Internal Medicine, Jizankai Tsuboi Hospital, Fukushima; The Tokyo Cooperative Oncology Group, Tokyo, Japan

**Keywords:** NSCLC, tegafur, CDHP, S-1

## Abstract

The purpose of this study was to evaluate the efficacy and safety of a novel oral anticancer fluoropyrimidine derivative, S-1, in patients receiving initial chemotherapy for unresectable, advanced non-small-cell lung cancer (NSCLC). Between June 1996 and July 1998, 62 patients with NSCLC who had not received previous chemotherapy for advanced disease were enrolled in this study. 59 patients (22 stage IIIB and 37 stage IV) were eligible for the evaluation of efficacy and safety. S-1 was administered orally, twice daily, after meals. 3 dosages of S-1 were prescribed according to body surface area (BSA) so that they would be approximately equivalent to 80 mg m^−2^day^−1^: BSA < 1.25 m^2^, 40 mg b.i.d.; BSA≥1.25 but <1.5 m^2^; 50 mg b.i.d., and BSA≥1.5 m^2^: 60 mg b.i.d. One cycle consisted of consecutive administration of S-1 for 28 days followed by a 2-week rest period, and cycles were repeated up to 4 times. The partial response (PR) rate of the eligible patients was 22.0% (13/59); (95% confidence interval: 12.3–34.7%). A PR was observed in 22.7% (5/22) of the stage IIIB patients and 21.6% (8/37) of the stage IV patients. The median response duration was 3.4 months (1.1–13.7 months or longer). Grade 4 neutropenia was observed in one of the 59 patients (1.7%). The grade 3 or 4 toxicities consisted of decreased haemoglobin level in 1.7% of patients (1/59), neutropenia in 6.8% (4/59), thrombocytopenia in 1.7% (1/59), anorexia in 10.2% (6/59), diarrhoea in 8.5% (5/59), stomatitis in 1.7% (1/59), and malaise in 6.8% (4/59), and their incidences were relatively low. There were no irreversible, severe or unexpected toxicities. The median survival time (MST) of all patients was 10.2 months (95% confidence interval: 7.7–14.5 months), and the one-year survival rate was 41.1%. The MST of the stage IIIB patients was 7.9 months, and that of the stage IV patients was 11.1 months. The one-year survival rates of the stage IIIB and IV patients were 30.7% and 47.4%, respectively. S-1 was considered to be an active single agent against NSCLC. Further study of S-1 with other active agents is warranted. © 2001 Cancer Research Campaignhttp://www.bjcancer.com


*British Journal of Cancer* (2001) **85**(7), 939–943
© 2001 Cancer Research Campaign
doi: 10.1054/ bjoc.2001.2031, available online at http://www.idealibrary.com on IDEAL

http://www.bjcancer.com

### Phase II study of S-1, a novel oral fluorouracil, in advanced non-small-cell lung cancer

**M Kawahara[1], K Furuse[1], Y Segawa[2], K Yoshimori[3], K Matsui[4], S Kudoh[5], K Hasegawa[6], H Niitani[7] and S-1 Cooperative Study Group (Lung Cancer Working Group)**

[1]Department of Internal Medicine, National Kinki Central Hospital for Chest Diseases, Osaka; [2]Department of Internal Medicine, National Shikoku Cancer Center Hospital, Matuyama; [3]Department of Respiratory Organs, Japan Anti-Tuberculosis Association Fukujuji Hospital, Tokyo; [4]Second Department of Internal Medicine, Osaka Prefectural Habikino Hospital, Osaka; [5]Fourth Department of Internal Medicine, Nippon Medical School, Tokyo; [6]Department of Internal Medicine, Jizankai Tsuboi Hospital Fukushima; [7]Study Director, The Tokyo Cooperative Oncology Group, Tokyo, Japan

**Summary** The purpose of this study was to evaluate the efficacy and safety of a novel oral anticancer fluoropyrimidine derivative, S-1, in patients receiving initial chemotherapy for unresectable, advanced non-small-cell lung cancer (NSCLC). Between June 1996 and July 1998, 62 patients with NSCLC who had not received previous chemotherapy for advanced disease were enrolled in this study. 59 patients (22 stage IIIB and 37 stage IV) were eligible for the evaluation of efficacy and safety. S-1 was administered orally, twice daily, after meals. 3 dosages of S-1 were prescribed according to body surface area (BSA) so that they would be approximately equivalent to 80 mg $m^{-2}$ $day^{-1}$: BSA < 1.25 $m^2$, 40 mg b.i.d.; BSA ≧ 1.25 but <1.5 $m^2$; 50 mg b.i.d., and BSA ≧ 1.5 $m^2$: 60 mg b.i.d. One cycle consisted of consecutive administration of S-1 for 28 days followed by a 2-week rest period, and cycles were repeated up to 4 times. The partial response (PR) rate of the eligible patients was 22.0% (13/59); (95% confidence interval: 12.3–34.7%). A PR was observed in 22.7% (5/22) of the stage IIIB patients and 21.6% (8/37) of the stage IV patients. The median response duration was 3.4 months (1.1–13.7 months or longer). Grade 4 neutropenia was observed in one of the 59 patients (1.7%). The grade 3 or 4 toxicities consisted of decreased haemoglobin level in 1.7% of patients (1/59), neutropenia in 6.8% (4/59), thrombocytopenia in 1.7% (1/59), anorexia in 10.2% (6/59), diarrhoea in 8.5% (5/59), stomatitis in 1.7% (1/59), and malaise in 6.8% (4/59), and their incidences were relatively low. There were no irreversible, severe or unexpected toxicities. The median survival time (MST) of all patients was 10.2 months (95% confidence interval: 7.7–14.5 months), and the one-year survival rate was 41.1%. The MST of the stage IIIB patients was 7.9 months, and that of the stage IV patients was 11.1 months. The one-year survival rates of the stage IIIB and IV patients were 30.7% and 47.4%, respectively. S-1 was considered to be an active single agent against NSCLC. Further study of S-1 with other active agents is warranted. © 2001 Cancer Research Campaign http://www.bjcancer.com

**Keywords**: NSCLC; tegafur; CDHP; S-1

Development of oral 5-fluorouracil (5-FU) antitumour drugs started in Japan in 1971, focusing on the fact that 5-FU acts in a time-dependent manner (Unemi et al, 1971), and, such drugs as uracil-tegafur (UFT) are currently being widely used in Japan to treat various types of cancer, including NSCLC, in combination with other drugs, such as cisplatin. Oral drugs enable patients to undergo treatment as outpatients, and they are suitable for maintaining patients' quality of life.

S-1 is a novel oral fluorpyrimidine derivative consisting of tegafur (FT) and 2 modulators, 5-chloro-2, 4-dihydroxypyridine (CDHP) and potassium oxonate (Oxo), in a molar ratio of 1:0.4:1 (Shirasaka et al, 1996) FT is a prodrug of 5-FU (Giller et al, 1967). CDHP is a reversible competitive inhibitor of dihydropyrimidine dehydrogenase (DPDase; EC1.3.1.2), an enzyme involved in the degradation of 5-FU (Tatsumi et al, 1987). Thus, the degradation of FT-derived 5-FU is efficiently inhibited by CDHP, and 5-FU remains in plasma and tumour tissue longer and at higher levels than when low-dose 5-FU is continuously infused intravenously. This has resulted in enhancement of the antitumour effect in an animal model (Shirasaka et al, 1996). The major toxicities of fluoropyrimidines are diarrhoea and mucositis (Vogelzang, 1984). Oxo is a reversible competitive inhibitor of orotate phosphoribosyltransferase (EC2.4.2.10), a phosphoenzyme for 5-FU, and is distributed at high levels in the gastrointestinal (GI) tract after oral administration, resulting in a reduction in GI toxicity caused by 5-FU (Shirasaka et al, 1993).

Based on the results of a phase I clinical trial, the maximum tolerated dose was concluded to be 75–100 mg $body^{-1}$, twice daily (as FT), and the dose-limiting factor was myelosuppression, especially leukopenia (Taguchi et al, 1994). In an early phase II clinical trial, the initial dosing schedule was 75 mg $body^{-1}$ b.i.d for 28 consecutive days followed by a 2-week rest period. However, the dose was reduced to 50 mg $body^{-1}$ b.i.d. because of skin rashes, severe myelosuppression, and diarrhoea. The response rate was 12.5% (5/40) in chemotherapy-naive patients. The major grade 3 or more toxicities were myelosuppression and gastrointestinal toxicity (Hino et al, 1996). When the actual administered doses were calculated according to body surface area (BSA), the rate of discontinuation of the drug because of toxicities was 71.4% (5/7) at 90 mg $m^{-2}$ $day^{-1}$. We therefore set the initial dose at 80 mg $m^{-2}$ $day^{-1}$ for this late phase II study.

To confirm the antitumour effect and safety of this drug, a multicentre late phase II clinical trial was conducted in 27 facilities in Japan.

*Received 24 November 2000*
*Revised 21 June 2001*
*Accepted 3 July 2001*

*Correspondence to:* M Kawahara

### PATIENTS AND METHODS

### Patient eligibility

Eligible patients were accrued between June 1996 and July 1998. The patients had histologically or cytologically confirmed stage IIIB or IV NSCLC and evidence of measurable disease (bone lesions were excluded from the evaluation). Prior therapy against NSCLC was an exclusion criterion. Other eligibility criteria included (1) age ≧ 20 years, but < 75 years, (2) Eastern Cooperative Oncology Group performance status of 0 to 2, (3) adequate bone marrow function (white blood cell count ≧ 4 × $10^9$ $l^{-1}$, but ≦ 12 × $10^9$ $l^{-1}$, haemoglobin ≧ 9.0 g $dl^{-1}$, and platelet count ≧ 100 × $10^9$ $l^{-1}$), (4) adequate liver function (total bilirubin ≦ 1.5 mg $ml^{-1}$, AST and ALT ≦ 100 IU $l^{-1}$, and alkaline phosphatase ≦ twice the upper normal limit), (5) adequate renal function (serum creatinine and blood urea nitrogen ≦ upper normal limit), (6) adequate pulmonary function ($PaO_2$ ≧ 70 Torr), (7) life expectancy ≧ 3 months, and (8) written informed consent.

Chest X-ray, chest computed tomography (CT), brain CT or magnetic resonance imaging (MRI), bone scintigraphy, and abdominal CT were used to stage the patients.

### Drug administration

S-1 was administered orally, twice daily, after meals. 3 doses of S-1 were selected according to body surface area (BSA) so that they would be approximately equivalent to 80 mg $m^{-2}$ $day^{-1}$: BSA < 1.25 $m^2$, 40 mg b.i.d.; BSA 1.25 $m^2$, but <1.5 $m^2$, 50 mg b.i.d.; and BSA ≧1.5 $m^2$, 60 mg b.i.d. One cycle consisted of consecutive administration of S-1 for 28 days followed by a 2-week rest period. The cycle could be repeated up to 4 times if no disease progression was detected. Patients in whom a complete response or partial response, as described below, was observed at the time of completion of the fourth cycle were transferred to the long-term administration study. Doses of the drug were adjusted according to haematological (WBC and platelet counts) and non-haematological toxicity. The dose was reduced by one level (20 mg $day^{-1}$) in patients whose body surface was 1.25 $m^2$ or more, with evidence of grade 3 or more haematologic toxicity (WBC < 2000 $\mu l^{-1}$ or platelet counts < 50 000 $\mu l^{-1}$) or grade 2 or more non-haematological toxicity (except alopecia and anorexia) during any cycle of administration. If recovery from such toxicities was confirmed at the reduced dose, administration at the reduced dose was continued. If a patient with body surface area less than 1.25 $m^2$, experienced the above toxicities, further treatment with S-1 was not done. If a rest period of more than 4 weeks was required, the patient was withdrawn from the study. Antiemetic drugs were not administered prophylactically. S-1 was provided by Taiho Pharmaceutical Co, Ltd (Tokyo, Japan).

### Measurements of study end points

Response was assessed in each cycle by clinical tumour measurements and documentation of the tumour size of measurable lesions.

The response to treatment of measurable lesions was evaluated in accordance with the criteria of the WHO (WHO Handbook for Reporting Results of Cancer Treatment, 1979). Safety was evaluated according to the Criteria for the Evaluation of the Clinical Effects of Solid Cancer Chemotherapy published by the Japan Society for Cancer Chemotherapy (Japan Society for Cancer Therapy, 1993). These criteria are almost the same as those published by the WHO, except for diarrhoea (see Table 2).

A complete response (CR) was defined as disappearance of all clinical and radiologic evidence of tumour for at least 4 weeks. A partial response (PR) required a ≧ 50% reduction in the sum of the products of the perpendicular diameters of all measurable lesions for at least 4 weeks. Progressive disease (PD) was defined as the appearance of an unequivocal new lesion or an increase of ≧ 25% in the sum of the products of the perpendicular diameters of any measured lesions No change (NC) or stable disease was a change insufficient for PR or PD for at least 4 weeks after the start of therapy. In addition, there could be no new lesions or increases in the size of any nonmeasurable lesions for CR, PR or NC.

Response duration was defined as the interval between the time that the criteria for response were first met and the time when disease progression was objectively documented. All of the judgements, including reported responses, were strictly inspected by extramural review based on CT scans, radiographs and magnetic resonance images. Overall survival time was measured as the interval between the start of S-1 treatment and the date of death or the date of the last follow-up. The Kaplan–Meier method was used to estimate overall survival curves (Kaplan and Meier, 1958).

Compliance was verified by patient interview. The ratio of the total dose actually administered to the scheduled dose was calculated for the first cycle.

The number of patients to be enrolled in this study was calculated as 60, the number required to refute the assumption that the 95% confidence interval would be 10% under conditions of $\alpha$ = 0.025 (one side) and $\beta$ = 0.1, assuming an expected response rate of 25%.

### RESULTS

62 patients were accured in this study, and 59 were assessable for response and toxicity. 3 patients were excluded as ineligible for the reasons described below. The drug was not administered to 1 patient because informed consent was withdrawn. In another patient, the primary lung cancer was found to be complicated by gastric cancer at the completion of the first cycle of chemotherapy. The other patient was judged to be ineligible due to the violation of one of the eligibility criteria (WBC count >12 × $10^9$ $l^{-1}$). Tumour

**Table 1** Patient characteristics ($n$ = 59)

| Characteristics | No. of Pts. |
|---|---|
| Male/female | 36/23 |
| Median age, years (range) | 64 (42–74) |
| Performance status | |
| 0 | 18 |
| 1 | 37 |
| 2 | 4 |
| Median initial dose, mg $m^{-2}$ (range) | 73.4 (64.1–79.5) |
| Stage of disease | |
| IIIB | 22 |
| IV | 37 |
| Histology | |
| Adenocarcinoma | 38 |
| Squamous cell carcinoma | 20 |
| Lage cell carcinoma | 1 |

*© 2001 Cancer Research Campaign*

response and toxicities were evaluated in the remaining 59 eligible patients. The characteristics of these 59 patients are listed in Table 1. 23 patients were female, representing 39% of all patients. Median age was 64 years, and age ranged from 42–74 years. Most of the patients had an ECOG performance status (PS) of 0 to 1, and only 4 patients (7%) were PS 2. 22 (37%) patients had stage IIIB disease, and the other 37 patients had stage IV disease. 38 (64%) of the 59 patients had adenocarcinoma, and 20 (34%) had squamous cell carcinoma.

The median follow-up period was 9.2 months (range, 1.6–32.7 months). The date of death was used as the date of the last follow up for patients who died.

### Antitumour activity

No CRs were observed in any of the 59 eligible patients, but a PR was observed in 13 patients, resulting in an overall response rate of 22.0% (95% confidence interval: 12.3–34.7%). The response rate among all 61 patients given the drug was 21.3% (95% confidence interval: 11.9–33.7%). Histologically, a PR was observed in 10 (26.3%) of the 38 patients with adenocarcinoma, 2 (10.0%) of the 20 patients with squamous cell carcinoma, and 1 patient with large cell carcinoma. According to clinical stage, a PR was observed in 22.7% (5/22) of the stage IIIB patients and 21.6% (8/37) of the stage IV patients. There was little difference in response rate between the stage IIIB and stage IV patients. The median response duration was 3.4 months (1.1–13.7 months or longer). As shown in Figure 1, the median survival time of all patients was 10.2 months (95% confidence interval: 7.7–14.5 months), and the 1-year survival rate was 41.1%. The median survival time of the stage IIIB patients was 7.9 months and that of the stage IV patients was 11.1 months. The 1-year survival rates of the stage IIIB and IV patients, were 30.7% and 47.4%, respectively. Although the survival of stage IV patients was longer than that of the stage IIIB patients, there was no statistically significant difference between them.

### Toxicity

The major toxicities during the study period are shown in Table 2. The incidences of the toxicities were evaluated in 59 eligible patients. Both haematological and non-haematological toxicities were mild. The toxicities reaching grade 3 or more consisted of decreased haemoglobin level in 1.7% of patients (1/59), neutropenia in 6.8% (4/59), and thrombocytopenia in 1.7% (1/59). The non-haematological grade 3 toxicities were anorexia in 10.2% (6/59), diarrhoea (grade 3: watery stools lasting more than 4 days) in 8.5% (5/59) and malaise in 6.8% (4/59). There were no grade 4 non-haematological toxicities. All of these toxicities were manageable, and the patients recovered when the drug was interrupted. There were no irreversible, severe, or unexpected toxicities.

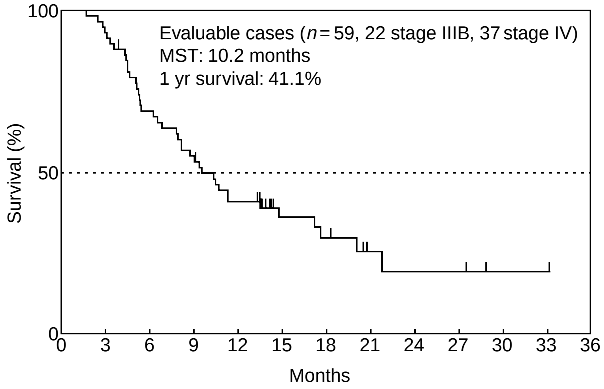

**Figure 1** Overall survival

**Table 2** Toxicity (*n* = 59)

| | Grade | | | | Grade 3–4 |
|---|---|---|---|---|---|
| | **1** | **2** | **3** | **4** | **(%)** |
| Haematological | | | | | |
| Leukopenia | 12 | 7 | 0 | 0 | |
| Neutropenia | 11 | 5 | 3 | 1 | 6.8 |
| Haemoglobin | 8 | 10 | 1 | 0 | 1.7 |
| Thrombocytopenia | 5 | 1 | 1 | 0 | 1.7 |
| Non-Haematological | | | | | |
| GOT (AST) | 6 | 2 | 0 | 0 | |
| GPT (ALT) | 3 | 3 | 0 | 0 | |
| Bilirubin | 3 | 1 | 0 | 0 | |
| Anorexia | 13 | 7 | 6 | 0 | 10.2 |
| Nausea & vomiting | 17 | 6 | 0 | 0 | |
| Diarrhoea*[a] | 7 | 3 | 5 | 0 | 8.5 |
| Stomatitis | 10 | 3 | 1 | 0 | 1.7 |
| Eruption | 5 | 3 | 0 | 0 | |
| Pigmentation | 10 | 3 | 0 | 0 | |
| Malaise | 5 | 5 | 4 | 0 | 6.8 |

*[a]Grade of diarrhoea (duration of days): Grade 1 muddy stool (2–3); Grade 2 watery stool (3–4); Grade 3 watery stool (≥5); Grade 4 hemorrhagic, dehydration.

### Dose intensity of S-1

Of the 10 patients who received only one cycle of chemotherapy, only 1 patient had a PR and 9 patients had a NC. 31 patients received 2 or more cycles. Of these, 16 patients received 3 cycles and 10 had 4 cycles. The reasons for only one cycle of treatment are PD in 13 patients, NC in 7 patients, request for another treatment in 1 patient and toxicities in 7 patients. The toxicities consisted of grade 3 thrombocytopenia in 1 patient, grade 2–3 diarrhoea in 3 patients and grade 2–3 anorexia in 3 patients.

116 cycles were administered to 59 patients. The dose was reduced in 6 patients because of toxicities. In the first cycle, 59 patients received 90.9% of the planned dose of S-1. The 31 patients in the second cycle received 95.3% of the planned dose of S-1, the 16 patients in the third cycle received 90.0%, and the 10 patients in the fourth cycle received 100%.

### DISCUSSION

Recent chemotherapeutic agents used as single-agent therapy to treat chemotherapy-naïve NSCLC include paclitaxel, docetaxel, vinorelbine, gemcitabine, and irinotecan, and the response rates have ranged from approximately 15–30% (Devita, 1997). In this study of S-1, responses were observed in 13 patients, with a response rate of 22.0% (13/59) and 95% CI of 12.3–34.7%. This

© 2001 Cancer Research Campaign 

shows that oral S-1 is an active first-line treatment for patients with NSCLC. Various 5-FU derivatives, such as tegafur (Ansfield et al, 1983), UFT (Keicho et al, 1986), and 5'-deoxy-5-fluorouridine (5′DFUR) (Hara, 1984; Niitani et al, 1985) have been developed to potentiate anticancer activity by preventing 5-FU degradation or sustain the concentration of 5-FU in the blood, but none of them have yielded a good response rate of more than 15% (Ota et al, 1988). However, rationally engineered and metabolically activated DPD inhibiting fluoropyrimidines (DIF) have recently been developed, as exemplified by S-1 and BOF-A2. BOF-A2, which has a 18% response rate (Nakai et al, 1994), is reported to be active against NSCLC. The mechanism of action is the same as that of S-1. There are several possible explanations for the enhanced activity of DIF such as S-1 or BOF-A2. In an in vitro study, S-1 had a higher therapeutic effect on human colon carcinoma implanted into nude rats than UFT did (Shirasaka et al, 1996). In vitro, CDHP has been shown to exert DPD inhibitory activity 180-fold higher than that of uracil, which has been confirmed to be a DPD inhibitor in the form of UFT (Tatsumi et al, 1987). Another point is that orally administered S-1 mimics prolonged continuous 5-FU infusion. A pharmacokinetic study of S-1 has been reported by Hirata et al (1999). They reported that there were no fluctuations in pharmacokinetics and no drug accumulation during a 28-day consecutive regimen of S-1, and that pharmacokinetic parameters of oral S-1 are almost the same to those of continuous i.v. infusion of 5-FU. Capecitabine is also capable of mimicking the mechanism of action of continuous infusion of 5-FU. This drug, not belonging to the DIF, is a prodrug that is metabolized to 5-FU upon absorption from the gastrointestinal tract. This drug is active against colorectal cancer and breast cancer (Blum et al, 1999; Cutsem et al, 2000). However, the activity of this drug against NSCLC has not been reported.

As regards haematological toxicity, grade 3 or more neutropenia was observed in only 4 patients (6.8%). Grade 3 anaemia and thrombocytopenia were observed only in 1 patient each (1.7%). The major non-haematological toxicities were anorexia (grade 3, 10.2%), diarrhoea (grade 3, 8.5%), and fatigue (grade 3, 6.8%), and they were mild and tolerable. The frequency of grade 3 or more adverse reactions was 2–4%, and there were no grade 4 adverse reactions. All of these toxicities are consistent with 5-FU-related toxicity. Oxo should protect against diarrhoea by selectively inhibiting 5-FU phosphorylation via orotate phosphoribosyltransferase in normal gastrointestinal tissue while minimizing its inhibition in tumour tissues (Shirasaka et al, 1993). The incidence of major toxicities associated with administration of UFT in phase II trials in various cancers have been 4% for leukopenia and 11% for diarrhoea ($n$ = 551) (Ota et al, 1988). The incidence of toxicities in our S-1 study was lower than observed for UFT.

116 cycles were administered to 59 patients, and the dose was reduced because of toxicities in only 6 patients. The high relative dose intensity of 90.0–100% during each cycle indicates that S-1 is easy to administer. This treatment is associated with fewer toxicities and outpatient treatment is feasible.

In this study, the median survival time of the stage IIIB patients was 7.9 months and that of the stage IV patients was 11.1 months. The survival time of stage IV patients seems longer than that of the stage IIIB patients, but the difference was not statistically significant. This may be due to small number of stage IIIB patients (22 patients).

At the time of this writing, S-1 has been approved in Japan for use in gastric cancer alone. Because of the limited indication for S-1, there have not been any combination chemotherapy trials of S-1 in NSCLC. Thus far, 3 phase II combination studies of another previously developed fluoropyrimidine derivative, UFT, plus cisplatin have been conducted in advanced NSCLC in Japan. In one trial by Nakai and Colleagues (Nakai et al, 1999), UFT was administered at 400 mg $m^{-2}$ on days 1 through 21 with cisplatin at 20 mg $m^{-2}$ on days 8 through 12. The response rate was 38.3% among 47 patients, and the MST was 12.8 months. In another study, by Yoshimori and Coworbers (Yoshimori et al, 1998), UFT was used on days 1 through 14 with cisplatin at 80 mg $m^{-2}$ on day 8. The response rate was 29% among 108 patients with a MST of 9.4 months. The other trial, by Ichinose et al, (1995), UFT 400 mg $m^{-2}$ on days 1 through 21 with cisplatin 80 mg $m^{-2}$ on day 8 was used. The response rate was 35% among 31 patients with median survival times of 11 months in the stage IIIB patients and 8 months in the stage IV patients. All trials reported an extremely low incidence of side effects, including neutropenia. The activity of UFT plus cisplatin is comparable to that of other cisplatin-based regimens, and the combination is well tolerated. These findings suggest that replacement of UFT by S-1 will further improve the response rate and survival will also improve. A study of S-1 plus cisplatin is planned.

In conclusion, our study indicates that S-1 is active in the treatment of NSCLC and provides a significant response rate with tolerable toxicity. The oral formulation and low incidence of adverse reactions permit treatment on an outpatient basis. S-1 is therefore considered to be a very convenient drug for patients. Further assessment in combination with other active agents is warranted.

### ACKNOWLEDGEMENTS

The authors are grateful to A Hamajima, T Kamizono, D Nozaki, N Nishi, N Fujimaki and H Yamashita (Taiho Pharmaceutical Co, Ltd, Tokyo, Japan) for their assistance in data collection and analysis. This study was supported by Taiho Pharmaceutical Co, Ltd, Tokyo, Japan.

### APPENDIX

Additional participating institutions and principal investigators: Hiroshi Isobe, Department of Respiratory Disease, National Sapporo Hospital, Sapporo; Toshihiro Nukiwa, Respiratory Oncology and Melecular Medicine, Institute of Development, Aging and Cancer, Tohoku University, Miyagi; Akira Yokoyama, Department of Internal Medicine, Niigata Cancer Center Hospital, Niigata; Hiroshi Sakai, Department of Respiratory Disease, Saitama Cancer Center, Saitama; Mitunori Hino, Department of Internal Medicine, Chiba-Hokusou Hospital, Nippon Medical School, Chiba; Takahiko Sugiura, Department of Pulmonary Disease, Aichi Cancer Center, Aichi; Hiromi Wada, Institute for Frontier Medical Sciences, Kyoto University, Kyoto; Syunichi Negoro, Department of Pulmonary Medicine, Osaka City General Hospital, Osaka; Minoru Takada, Department of Respiratory Disease, Izumisano Municipal Hospital, Osaka; Soichiro Yokota, Department of Internal Medicine, Toneyama National Hospital, Osaka; Yoshiki Takada, Department of Radiology, Hyogo Medical Center for Adult Diseases, Hyogo; Hiroshi Ueoka, Second Department of Internal Medicine, Okayama University School of Medicine, Okayama; Michio Yamakido, Second Department of Internal Medicine, Hiroshima University School of Medicine;

 © 2001 Cancer Research Campaign

Hiroshima; Nobuyuki Hara, Research Institute for Diseases of The Chest, Research Institute for Diseases of The Chest, Faculty of Medicine, Kyushu University, Fukuoka; Yukito Ichinose, Department of Chest Surgery, National Kyushu Cancer Center, Fukuoka; Hidehiko Yamamoto, Department of Pulmonary Disease, Aso Cement Iizuka Hospital, Fukuoka.

### REFERENCES

Ansfield FJ, Kallas GJ and Singson JP (1983) Phase I-II studies of oral Tegafur (Ftorafur). *J Clin Oncol* **1**: 107–110

Blum JL, Jones SE, Buzdar AU, LoRusso PM, Kuter I, Vogel C, Osterwalder B, Burger H, Brown CS and Griffin T (1999) Multicenter phase II study of capecitabine in paclitaxel-refractory metastatic breast cancer. *J Clin Oncol* **17**: 485–493

Cutsem EV, Findlay M, Osterwalder B, Kocha W, Dalley D, Pazdur R, Cassidy J, Dirix L, Twelves C, Allman D, Seitz JF, Schölmerich J, Burger HU and Verweij J (2000) Capecitabine, an oral fluoropyrimidine carbamate with substantial activity in advanced colorectal cancer: results of a randomized phase II study. *J Clin Oncol* **18**: 1337–1345

Devita Jr, VT (1997) *Principles & Practice of Oncology*, Fifth Edition, pp 858–911 Lippincott-Raven Publishers

Giller SA, Zhuk Ra and Lidak MY (1967) Analogs of pyrimidine nucleosides I. $N_1$-(α-tetrahydrofuryl) derivatives of natural pyrimidine bases and their antimetabolites (in Russian). *Dokl Akad Nauk SSSR* **176**: 332–335

Hara Y (1984) 5′-deoxy-5-fluorouridine enzymatic activation from the masked compound to 5-fluorouracil in human malignant tissues. *Jpn J Cancer Chemother* **11**: 2133–2143 (in Japanese with English Abstract)

Harris BE, Song R, Soong S and Diasio RB (1990) Relationship between dihydropyrimidine dehydrogenase activity and plasma 5-fluorouracil levels with evidence for circadian variation of enzyme activity and plasma drug levels in cancer patients receiving 5-fluorouracil by protracted continuous infusion. *Cancer Res* **50**: 197–201

Heggie GD, Sommadossi J-P, Cross DS, Huster WJ and Diasio RB (1987) Clinical pharmacokinetics of 5-fluorouracil and its metabolites in plasma, urine, and bile. *Cancer Res* **47**: 2203–2206

Hino M, Kudoh S, Furuse K, Hasegawa K, Takada M, Ichinose Y, Sugiura T and Niitani H (1996) Early phase II Study of S-1 in Patients with Non-Small-Cell Lung Cancer *Ann Oncol* **7**(Suppl.5): 503

Hirata K, Horikoshi N, Aiba K et al (1999) pharmacokinetic study of S-1, a novel oral fluorouracil antitumor drug. *Clinical Cancer Research* **5**: 2000–2005

Ichinose Y, Takanashi N, Yano T, Asoh H, Yokoyama H, Tayama K, Hara N and Ohta M (1995) A phase II trial of oral tegafur and uracil plus cisplatin in patients with inoperable nonsmall cell lung cancer. *Cancer* **75**: 2677–2680

Ikenaka k, Shirasaka T, Kitano S and Fujii S (1979) Effect of uracil on metabolism of 5-fluorouracil in vitro. *Jpn J Cancer Res (Gann)* **70**: 353–359

Japan Society for Cancer Therapy (1993) Criteria for the evaluation of the clinical effects of solid cancer chemotherapy. *J Jpn Soc Cancer Ther* **28**: 101–130

Kaplan EL and Meier P (1958) Nonparametric estimation from incomplete observations. *Am Stat Assoc* **53**: 457–481

Keicho N, Saijo N, Shinkai T, Eguchi K, Sasaki Y, Tamura T, Sakurai M, Sano T and Hoshi A (1986) Phase II study of UFT in patients with advanced non-small cell lung cancer. *Jpn J Clin Oncol* **16**: 143–146

Lokich JJ, Ahlgren JD, Gullo JJ, Philips JA and Fryer JG (1989) A prospective randomized comparison of continuous infusion fluorouracil with a conventional bolus schedule in metastatic colorectal carcinoma: a mid-Atlantic oncology program study. *J Clin Oncol* **7**: 425–432

Moynihan T, Hansen R, Anderson T, Quebbeman E, Beatty P, Ausman R, Ritch P, Chitambar C and Vukelich M (1988) Continuous 5-fluorouracil infusion in advanced gastric carcinoma. *Am J Clin Oncol* **11**: 461–464

Nakai Y, Furuse K, Ohta M, Yamaguchi Y, Fujii M, Asakawa M, Fukuoka M, Yoshida K and Niitani H (1994) Efficacy of a new 5-fluorouracil derivative, BOF-A2, in advanced non-small cell lung cancer. A multi-center phase II study. *Acta Oncologica* **33**: 523–526

Nakai Y, Saito J, Koinumaru S and Saijo Y (1999) A phase II trial of oral UFT and cisplatin in patients with advanced non-small cell lung cancer. *Proc Am Soc Clin Oncol* **18**(Abstr 1866): 483a

Niitani H, Kimura K, Saito T, Nakao I, Abe O, Urushizaki I, Ohta K, Yoshida Y, Kimura T, Kurihara M, Takeda C, Taguchi T, Terasawa T, Tominaga K, Furue H, Wakui A and Ogawa N (1985) Phase II study of 5′-deoxy-5-fluorouridine(5′DFUR) on patients with malignant cancer-Multi-institutional cooperative study. *Jpn J Cancer Chemother* **12**(in Japanese with English Abstract): 2044–2051

Ota K, Taguchi T and Kimura K (1988) Report on nationwide pooled data and cohort investigation in UFT phase II study. *Cancer Chemother Pharmacol* **22**: 333–338

Richards F, Perry D, Goutsou M, Modeas C, Muchmore E, Rege V, Chahinian AP, Hirsh V, Poiesz B and Green MR (1991) Chemotherapy with 5-fluorouracil[5-FU] and cisplatin or 5-FU, cisplatin, and vinblastine for advanced non-small cell lung cancer. A randomized phase II study of the Cancer and Leukemia Group B. *Cancer* **67**: 2974–2979

Seifert P, Baker LH, Reed ML and Vaitkevicius VK (1975) Comparison of continuously infused 5-fluorouracil with bolus injection in treatment of patients with colorectal adenocarcinoma. *Cancer* **36**: 123–128

Shirasaka T, Shimamoto Y and Fukushima M (1993) Inhibition by oxonic acid of gastrointestinal toxicity of 5-fluorouracil without loss of its antitumor activity in rats. *Cancer Res* **53**: 4004–4009

Shirasaka T, Nakano K, Takechi T, Satake H, Uchida J, Fujioka A, Saito H, Okabe H, Oyama K, Takeda S, Unemi N and Fukushima M (1996a) Antitumor activity of 1M tegafur-0.4M 5-chloro-2,4-dihydroxypyridine-1M potassium oxonate (S-1) against human colon carcinoma orthotopically implanted into nude rats. *Cancer Res* **56**: 2602–2606

Shirasaka T, Shimamoto Y, Ohshimo H, Yamaguchi M, Kato T, Yonekura K and Fukushima M (1996b) Development of a novel form of an oral 5-fluorouracil derivative (S-1) directed to the potentiation of the tumor selective cytotoxicity of 5-fluorouracil by two biochemical modulators. *Anti-Cancer Drugs* **7**: 548–557

Taguchi T, Inuyama Y, Kanamaru R, Hasegawa K, Akazawa S, Niitani H, Furue H, Kurihara M, Ota K, Suga S, Ariyoshi Y, Takai S, Shimoyama T, Toge T, Takashima S, Sugimachi K, Hara Y, Fujita H, Kimura K, Saito T, Tsukagoshi S and Nakao I (1997) Phase I study of S-1. *Jpn J Cancer Chemother* **24**(in Japanese with English Abstract): 2253–2264

Tatsumi K, Fukushima M, Shirasaka T and Fujii S (1987) Inhibitory effects of pyrimidine, barbituric acid and pyridine derivatives on 5-fluorouracil degradation in rat liver extracts. *Jpn J Cancer Res (Gann)* **78**: 748–755

Unemi N, Harima K, Daidai Y, Fujiwara M and Fujii S (1971) Experimental studies of N1-(2′-tetrahydrofuryl)-5-fluorouracil(FT-207). *Gan No Rinsho* **17**: 731–742

Vogelzang NJ (1984) Continuous infusion chemotherapy: a critical review. *J Clin Oncol* **2**: 1289–1304

Weiden PL, Einstein AB and Rudolph RH (1985) Cisplatin bolus and 5-FU infusion chemotherapy for non-small cell lung cancer. *Cancer Treat Rep* **69**: 1253–1255

World Health Organization (1979) WHO Handbook for Reporting Results of Cancer Treatment. *WHO Offset Publ Geneva* No. 48

Yoshimori K, Yano T, Yoneda S, Kuba M, Ichinose Y, Kudoh S and Niitani H (1998) A phase II trial of UFT plus cisplatin in patients with advanced non-small cell lung cancer. *Proc Am Soc Clin Oncol* **17**(Abstr 1803): 469a

© 2001 Cancer Research Campaign

## References

[PDF_00481] Ansfield F. J., Kallas G. J., Singson J. P. (1983). Phase I-II studies of oral tegafur (ftorafur).. J Clin Oncol.

[PDF_00483] Blum J. L., Jones S. E., Buzdar A. U., LoRusso P. M., Kuter I., Vogel C., Osterwalder B., Burger H. U., Brown C. S., Griffin T. (1999). Multicenter phase II study of capecitabine in paclitaxel-refractory metastatic breast cancer.. J Clin Oncol.

[PDF_00494] Giller S. A., Zhuk R. A., Lidak M. Iu (1967). Analogi pirimidinnukleozidov. I. N1-(alpha-furanidil'nye)-proizvodnye prirodnykh pirimidinovykh osvanii i ikh antimetabolitov.. Dokl Akad Nauk SSSR.

[PDF_00498] Hara Y. (1984). [5'-Deoxy-5-fluorouridine enzymatic activation from the masked compound to 5-fluorouracil in human malignant tissues].. Gan To Kagaku Ryoho.

[PDF_00501] Harris B. E., Song R., Soong S. J., Diasio R. B. (1990). Relationship between dihydropyrimidine dehydrogenase activity and plasma 5-fluorouracil levels with evidence for circadian variation of enzyme activity and plasma drug levels in cancer patients receiving 5-fluorouracil by protracted continuous infusion.. Cancer Res.

[PDF_00506] Heggie G. D., Sommadossi J. P., Cross D. S., Huster W. J., Diasio R. B. (1987). Clinical pharmacokinetics of 5-fluorouracil and its metabolites in plasma, urine, and bile.. Cancer Res.

[PDF_00512] Hirata K., Horikoshi N., Aiba K., Okazaki M., Denno R., Sasaki K., Nakano Y., Ishizuka H., Yamada Y., Uno S. (1999). Pharmacokinetic study of S-1, a novel oral fluorouracil antitumor drug.. Clin Cancer Res.

[PDF_00514] Ichinose Y., Takanashi N., Yano T., Asoh H., Yokoyama H., Tayama K., Hara N., Ohta M. (1995). A phase II trial of oral tegafur and uracil plus cisplatin in patients with inoperable nonsmall cell lung cancer.. Cancer.

[PDF_00517] Ikenaka K., Shirasaka T., Kitano S., Fujii S. (1979). Effect of uracil on metabolism of 5-fluorouracil in vitro.. Gan.

[PDF_00523] Keicho N., Saijo N., Shinkai T., Eguchi K., Sasaki Y., Tamura T., Sakurai M., Sano T., Hoshi A. (1986). Phase II study of UFT in patients with advanced non-small cell lung cancer.. Jpn J Clin Oncol.

[PDF_00526] Lokich J. J., Ahlgren J. D., Gullo J. J., Philips J. A., Fryer J. G. (1989). A prospective randomized comparison of continuous infusion fluorouracil with a conventional bolus schedule in metastatic colorectal carcinoma: a Mid-Atlantic Oncology Program Study.. J Clin Oncol.

[PDF_00530] Moynihan T., Hansen R., Anderson T., Quebbeman E., Beatty P., Ausman R., Ritch P., Chitambar C., Vukelich M. (1988). Continuous 5-fluorouracil infusion in advanced gastric carcinoma.. Am J Clin Oncol.

[PDF_00533] Nakai Y., Furuse K., Ohta M., Yamaguchi Y., Fujii M., Asakawa M., Fukuoka M., Yoshida K., Niitani H. (1994). Efficacy of a new 5-fluorouracil derivative, BOF-A2, in advanced non-small cell lung cancer. A multi-center phase II study.. Acta Oncol.

[PDF_00540] Niitani H., Kimura K., Saito T., Nakao I., Abe O., Urushizaki I., Ohta K., Yoshida Y., Kimura T., Kurihara M. (1985). [Phase II study of 5'-deoxy-5-fluorouridine (5'-DFUR) in patients with malignant cancer--a multi-institutional cooperative study].. Gan To Kagaku Ryoho.

[PDF_00546] Ota K., Taguchi T., Kimura K. (1988). Report on nationwide pooled data and cohort investigation in UFT phase II study.. Cancer Chemother Pharmacol.

[PDF_00549] Richards F., Perry D. J., Goutsou M., Modeas C., Muchmore E., Rege V., Chahinian A. P., Hirsh V., Poiesz B., Green M. R. (1991). Chemotherapy with 5-fluorouracil (5-FU) and cisplatin or 5-FU, cisplatin, and vinblastine for advanced non-small cell lung cancer. A randomized phase II study of the cancer and leukemia group B.. Cancer.

[PDF_00554] Seifert P., Baker L. H., Reed M. L., Vaitkevicius V. K. (1975). Comparison of continuously infused 5-fluorouracil with bolus injection in treatment of patients with colorectal adenocarcinoma.. Cancer.

[PDF_00560] Shirasaka T., Nakano K., Takechi T., Satake H., Uchida J., Fujioka A., Saito H., Okabe H., Oyama K., Takeda S. (1996). Antitumor activity of 1 M tegafur-0.4 M 5-chloro-2,4-dihydroxypyridine-1 M potassium oxonate (S-1) against human colon carcinoma orthotopically implanted into nude rats.. Cancer Res.

[PDF_00565] Shirasaka T., Shimamato Y., Ohshimo H., Yamaguchi M., Kato T., Yonekura K., Fukushima M. (1996). Development of a novel form of an oral 5-fluorouracil derivative (S-1) directed to the potentiation of the tumor selective cytotoxicity of 5-fluorouracil by two biochemical modulators.. Anticancer Drugs.

[PDF_00557] Shirasaka T., Shimamoto Y., Fukushima M. (1993). Inhibition by oxonic acid of gastrointestinal toxicity of 5-fluorouracil without loss of its antitumor activity in rats.. Cancer Res.

[PDF_00570] Taguchi T., Inuyama Y., Kanamaru R., Hasegawa K., Akazawa S., Niitani H., Furue H., Kurihara M., Ota K., Suga S. (1997). [Phase I study of S-1. S-1 Study Group].. Gan To Kagaku Ryoho.

[PDF_00575] Tatsumi K., Fukushima M., Shirasaka T., Fujii S. (1987). Inhibitory effects of pyrimidine, barbituric acid and pyridine derivatives on 5-fluorouracil degradation in rat liver extracts.. Jpn J Cancer Res.

[PDF_00578] Unemi N., Harima K., Daidai Y., Fujihara H., Fujii S. (1971). [Experimental studies on antineoplastic action of N 1 -(2'-tetrahydrofuryl)-5-fluorouracil (FT-207)--effect on experimental tumors].. Gan No Rinsho.

[PDF_00487] Van Cutsem E., Findlay M., Osterwalder B., Kocha W., Dalley D., Pazdur R., Cassidy J., Dirix L., Twelves C., Allman D. (2000). Capecitabine, an oral fluoropyrimidine carbamate with substantial activity in advanced colorectal cancer: results of a randomized phase II study.. J Clin Oncol.

[PDF_00580] Vogelzang N. J. (1984). Continuous infusion chemotherapy: a critical review.. J Clin Oncol.

[PDF_00582] Weiden P. L., Einstein A. B., Rudolph R. H. (1985). Cisplatin bolus and 5-FU infusion chemotherapy for non-small cell lung cancer.. Cancer Treat Rep.

